# Comparative analysis of the microbial composition of three packaged sliced dry-cured hams from a Chinese market

**DOI:** 10.3389/fmicb.2025.1531005

**Published:** 2025-03-05

**Authors:** Xin Luo, Ying Shen

**Affiliations:** Guizhou Academy of Testing and Analysis, Guizhou Academy of Sciences, Guiyang, China

**Keywords:** packaged sliced dry-cured hams, Serrano prosciutto, Jinhua prosciutto, Xuanwei prosciutto, microbial composition

## Abstract

Ham, a widely consumed and culturally significant food, undergoes fermentation and aging processes that contribute to its distinctive flavor and texture. These processes are influenced by a complex interplay of microbial communities, which vary by the production region. Understanding these microbial dynamics can provide insights into flavor development and quality improvements in ham. In this study, the microbial communities found in ham produced in three distinct regions were compared, revealing that bacteria have a more dominant role in shaping the overall microbiota than fungi. Notably, each type of ham exhibited a unique microbial profile, although those from similar regions shared more similar profiles. Specific bacterial biomarkers were identified for each regional ham: *Lactobacillus* and *Tetragonococcus* in Serrano prosciutto, *Odoribacter*, *Alistipes*, *Staphylococcus*, and *Akkermansia* in Jinhua prosciutto, and *Pseudomonas*, *Blautia*, and *Bacteroides* in Xuanwei prosciutto. The microbial network analysis identified closer associations between microorganisms in the domestically produced Chinese hams than in the Spanish ham, suggesting limited foreign microbial invasions that contributed to a richer, more stable flavor. These findings offer new insights into how microbial interactions shape the development of flavor and quality in ham and clarify future strategies for improving the production process by leveraging microbial communities.

## 1 Introduction

Ham is a flavorful food with a rich history, cherished by the Chinese people and appreciated globally. During the 13th to 15th century, ham processing technology was introduced to Europe by Marco Polo, which profoundly influenced ham production outside of China and gave rise to diverse regional flavors (Zhou and Zhao, 2007). As ham processing continued to develop in various regions, foods with regional flavors gradually emerged, such as Serrano ham, Jinhua ham, and Xuanwei ham.

In the traditional ham-making process, fermentation is essential, and the process temperature, humidity, and strains of microorganisms collectively influence the taste of the final product (Bosse et al., 2018). Microorganisms, as the primary agents in the fermentation process, play a crucial role in flavor development (Yang et al., 2020). In traditional research, the composition of the microbial community in ham is mainly identified through microscopic observation and microbial cultivation, which can result in incomplete and inaccurate identification (Jiang et al., 2024). With the development of high-throughput sequencing technology, amplicon sequencing offers expanded avenues for exploring microbial diversity. Commonly employed methods include 16S rRNA gene sequencing for bacteria and Internal Transcribed Spacer (ITS) gene sequencing for fungi, which is extensively applied in environmental monitoring, gut microbiota studies, and other microbial ecology research (Morgan et al., 2017; Callahan et al., 2019). The existing research on the microbial community in ham showed that the bacterial composition of Norden ham is mainly dominated by Firmicutes, Proteobacteria, Actinobacteria, and Bacteroidetes, while Xuanwei ham is dominated by mold, *Staphylococcus*, and *Micrococcus* (Zhang, 2014; Zou et al., 2020). However, there is limited research comparing the microbial composition and internal microbial interactions among Xuanwei, Serrano, and Jinhua ham.

This study used next-generation sequencing technology to analyze the bacteria and fungi present in three commercially available hams, with the aim of clarifying the microbial composition, biomarkers, and interactions within their respective communities. These findings provide a systematic theoretical framework for understanding the formation of distinct flavors in these hams and enhancing their overall quality.

## 2 Materials and methods

### 2.1 Sampling

Slices of three types of vacuum-packed, ready-to-eat cured prosciutto were purchased from a market: Serrano prosciutto (SRP), Jinhua prosciutto (JHP), and Xuanwei prosciutto (XWP). The labels indicated that these hams originated from Spain; Jinhua, China; and Xuanwei, China, respectively, and had been cured for over a year. After purchasing, an appropriately sized sample obtained from the same area of each type of prosciutto was collected under a sterile hood and used as the basis for sequencing. Three samples of each type of ham were randomly selected for the experiment and stored at −80°C until used.

### 2.2 DNA extraction and sequencing

The samples were stored at −80°C and transported on dry ice. Total DNA was extracted using a DNA extraction kit, and the purity and concentration of the DNA were assessed using 1% agarose gel electrophoresis and a NanoDrop One. The 16S rRNA gene V3–V4 variable region was amplified using the forward primer 5′-ACTCCTACGGGAGGCAGCA-3′ and the reverse primer 5′-GGACTACHVGGGTWTCTAAT-3′, and the rRNA gene ITS1 region was amplified using the forward primer 5′-CTTGGTCATTTAGAGGAAGTAA-3′ and the reverse primer 5′-GCTGCGTTCTTCATCGATGC-3′. The PCR reaction program consisted of an initial denaturation at 98°C for 1 min; 30 cycles at 98°C for 10 s, 50°C for 30 s, and 72°C for 30 s; and a final extension at 72°C for 5 min. The PCR products were subjected to electrophoresis on a 2% agarose gel and purified using a DNA purification and recovery kit. The library was prepared using an NEB Next® Ultra™ II FS DNA PCR-free library prep kit and sequenced on an Illumina NovaSeq 6,000 platform by Biomarker Technologies Co., Ltd., with paired-end sequencing (PE250, 250 bp).

### 2.3 Composition and diversity analysis

After the data were obtained, Python scripts were used to split the library and remove barcode and primer sequences. Quality control of the raw data was performed using Trimmomatic (v0.33) (Bolger et al., 2014), and primer sequences were identified and removed using cutadapt (v1.9.1) (Martin, 2011). USEARCH was used for paired-end merging, and UCHIME (v 8.1) (Edgar et al., 2011; Edgar, 2013) was used to remove chimeras. Species annotation was performed and a feature table was generated using the Quantitative Insights into Microbial Ecology (v202202) pipeline (Bolyen et al., 2019).

After normalization, Amplicon Sequence Variants (ASVs) with a total abundance of <30 and <2 occurrences were removed. The *α*- and *β*-diversity indices were calculated using the vegan package (Oksanen et al., 2013) with the species annotation database Silva (v138.1) (Quast et al., 2013). Visualization was performed using RStudio (v4.0.3), and Venn diagrams and petal plots were generated by the VennDiagram package. Differences in the α-diversity between groups were tested using Wilcoxon tests with the ggsignif package, with *p* ≤ 0.05 indicating significant differences and *p* ≤ 0.01 indicating highly significant differences. LEfSe analysis was performed separately using the microeco R package (Liu C. et al., 2021).

### 2.4 Network interaction analysis

After normalization, ASVs with a total frequency of <0.2 were removed. Correlation analysis was performed based on Spearman’s rank correlation, and values with *p* > 0.05 and a correlation coefficient < 0.6 were eliminated. The interactions within bacterial and fungal microbiomes were analyzed separately. The interactions between the microbiomes were analyzed using the WGCNA (version 1.72.5), psych (version 2.4.6.26), and igraph (version 2.0.3) R packages (Langfelder and Horvath, 2008; Csardi, 2013; Revelle and Revelle, 2015). Gephi software (version 0.1.0) (Bastian et al., 2009) was used for visualization.

## 3 Results

### 3.1 Analysis of the microbial diversity

**Figure 1:**
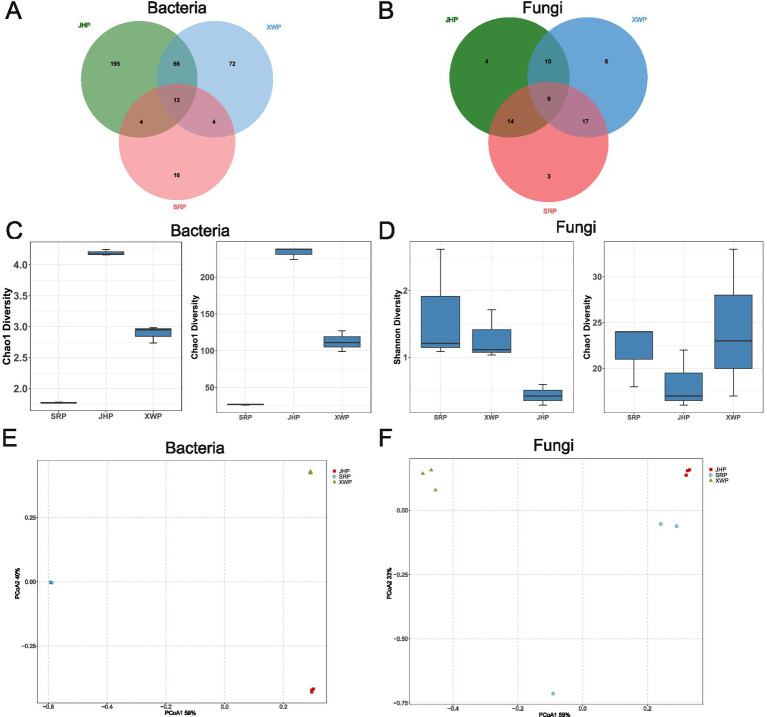
Diversity analysis of fungal and bacterial communities in different types of prosciuttos. **(A,B)** The shared and unique characteristics among bacteria **(A)** and fungi **(B)** in different groups. **(C,D)** Analysis of the *α*-diversity of bacteria **(C)** and fungi **(D)** in each group using Shannon and Chao1 indices. **(E,F)** Principal coordinates analysis based on Bray–Curtis distances of bacteria **(E)** and fungi **(F)** in the three types of ham.

Microbial diversity is an important means of understanding the microbial community in food. The JHP group and XWP group had the highest number of bacteria in common but shared the lowest number of fungal types. The three groups shared 13 bacterial and 9 fungal microbial communities (Figures 1A,B). The Shannon and Chao1 diversity indices are important indicators for measuring microbial richness and evenness. The uniformity of the bacteria was highest in the JHP group, while the uniformity of the fungi was highest in the SRP group. The number of bacterial species was highest in the JHP group, and the uniformity of the fungi was highest in the XWP group (Figures 1C,D). The diversity analysis results of the data show that there were significant differences in bacteria and fungi among the different types of ham (Figures 1E,F).

### 3.2 Composition of and differences in bacteria

**Figure 2:**
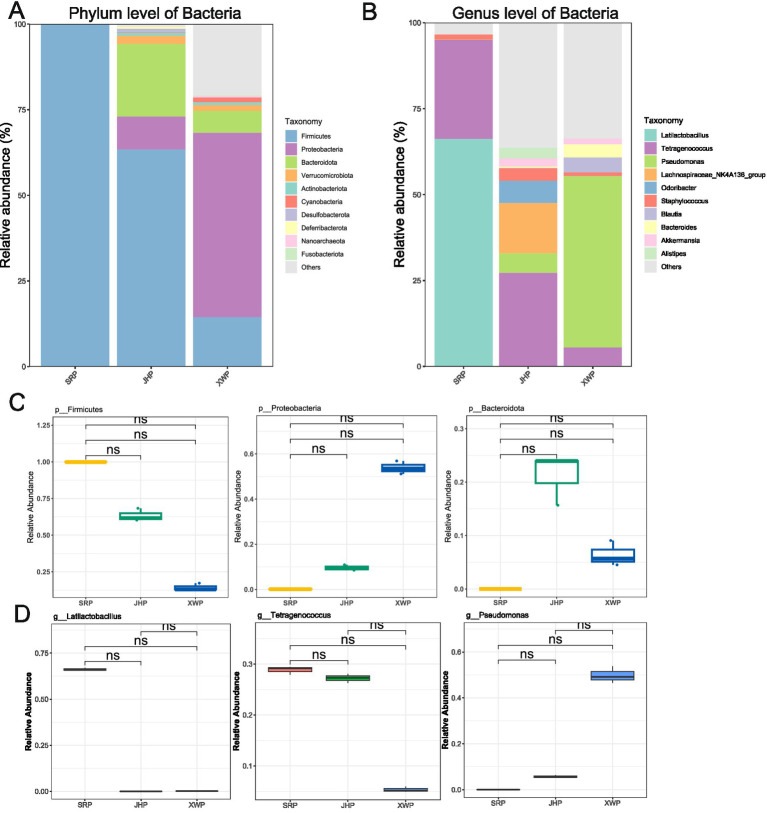
Analysis of the composition and abundance of bacteria at the phylum and genus levels among the three hams, and differences in the top three phyla and genera. **(A,C)** Analysis of the bacterial composition and ranking of the three different types of ham at the phylum level. **(B,D)** Analysis of differences in the bacterial composition and ranking of the three different types of ham at the genus level. Wilcoxon significance tests were performed between pairs.

Different types of ham are greatly affected by fermentation process parameters; thus, the microbial community is also greatly affected. Bacteria, as an important component of the microbial community, has a crucial role in the fermentation process. The ham in the SRP and JHP groups was dominated by Firmicutes, while the XWP group was primarily associated with Proteobacteria, indicating that different types of ham exhibit variations in microbial composition (Figure 2A). At the genus level, the species composition was further elucidated, with *Lactobacillus* and *Tetragonococcus* accounting for over 90% of the bacteria in the SRP group. In contrast, *Tetragonococcus* and the *Lachnospiraceae NK4A136* group were dominant in the JHP group. In the XWP group, however, *Pseudomonas* was the predominant genus (Figure 2B). These significant differences in composition contribute to the distinct flavors of the ham. Although there were notable differences in specific phyla and genera between groups, no significant differences were observed, which may be related to the number of samples (Figures 2C,D).

### 3.3 Composition of and differences in fungi

**Figure 3:**
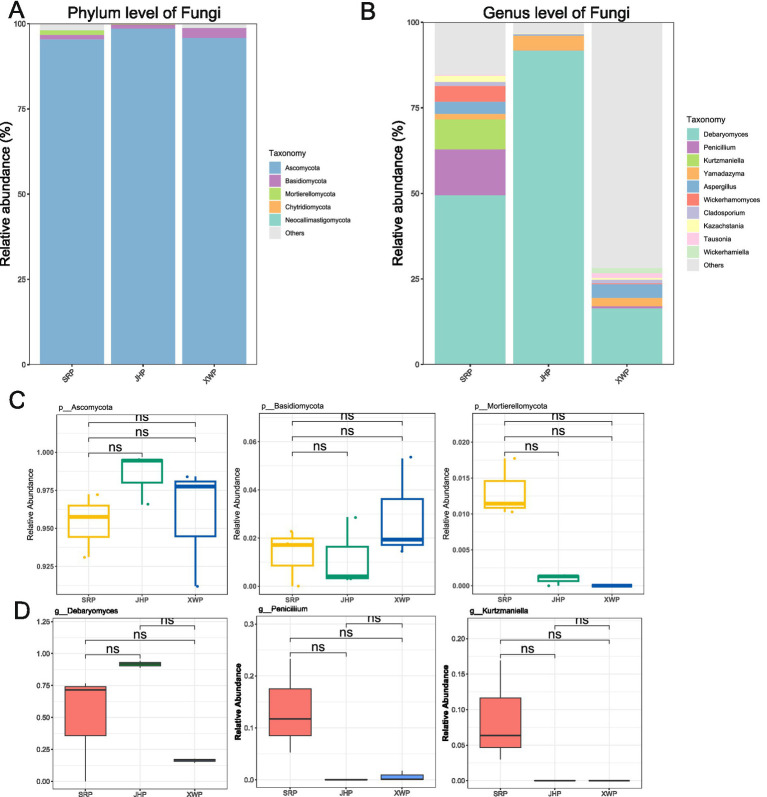
Analysis of the composition and abundance of fungi at the phylum and genus levels, and phyla and genera among the three hams. **(A,C)** Analysis of the fungal composition and ranking of the three different types of ham at the phylum level. **(B,D)** Analysis of the fungal composition and ranking of the three types of ham at the genus level. Wilcoxon significance tests were performed between pairs.

Fungi, as an important component of microbial communities, also contribute to the formation of flavors. Overall, Ascomycota was the main fungal phylum among the three types of ham (Figure 3A). At the genus level, *Debarytomyces* was predominant, and there were differences in the composition and abundance of fungi in the three types of ham. The formation of flavor by fungi may be due to changes in the proportions of the fungal composition (Figures 3C,D).

### 3.4 Linear discriminant analysis effect size

**Figure 4:**
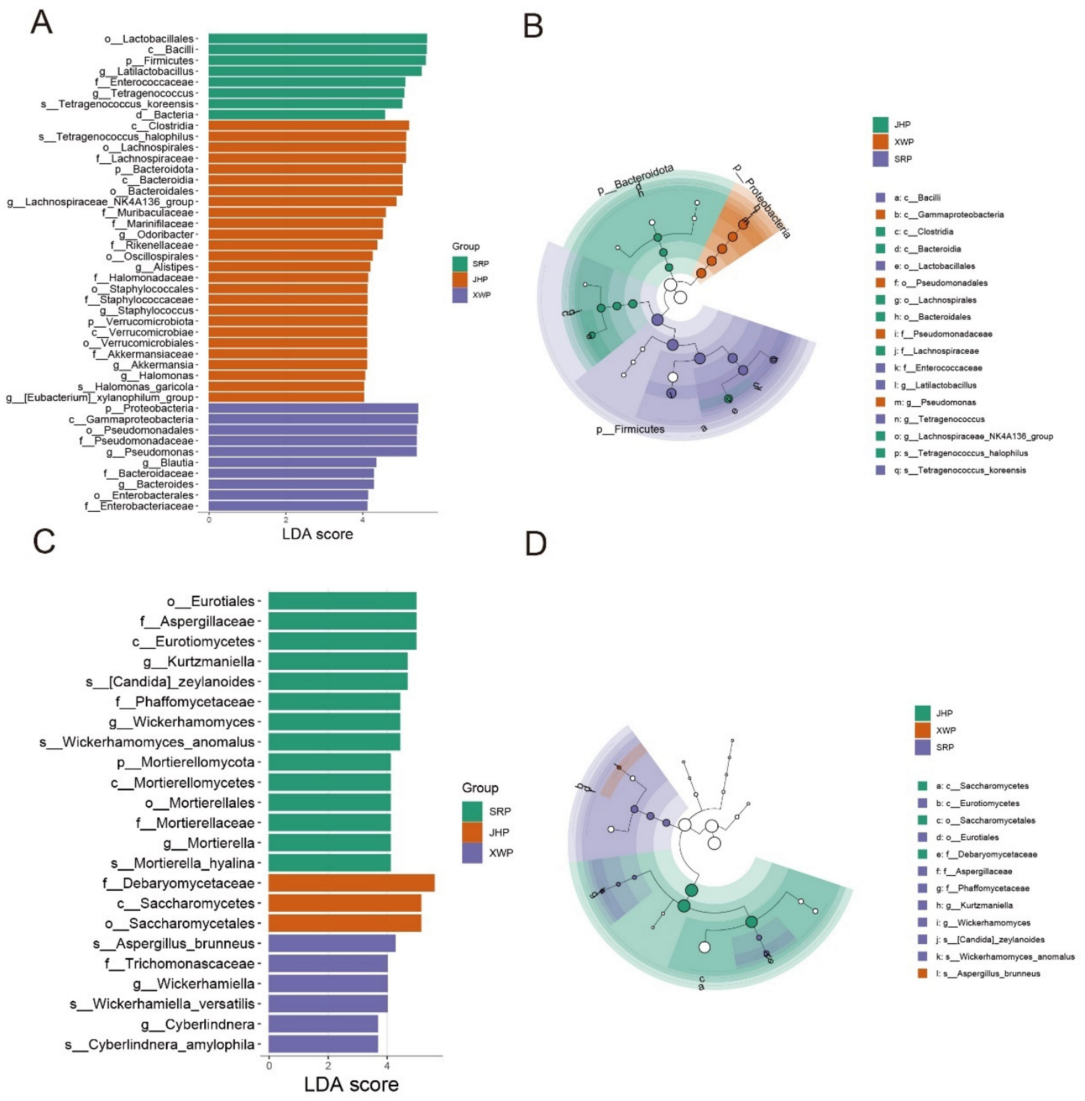
Biomarker screening of fungi and bacteria in different hams. **(A,B)** Biomarkers based on Linear Discriminant Analysis (LDA) > 4 in different bacterial taxa of the three hams and the associated cladogram. **(C,D)** Biomarkers based on LDA > 3 in different fungal taxa of the three hams and the associated cladogram.

Biomarker screening based on LEfSe is an important analytical method for analyzing the formation of ham flavor among different types of ham. Within the bacterial community, the SRP group included genera from the Firmicutes, specifically *Lactobacillus* and *Tetragonococcus*, as biomarkers (Figures 4A,B). The JHP group included members of Bacteroidota, such as *Odorobacter*, *Alistipes*, *Staphylococcus*, *Akkermansia*, *Halomonas*, and the *xylanophyllum* group, as biomarkers. In the XWP group, *Pseudomonas*, *Blautia*, and *Bacteroides* from Proteobacteria were identified as key biomarkers. The biomarker screening of fungi revealed that the SRP group was characterized by the genera *Kurtzmaniella*, *Wickerhamomyces*, and *Mortierella* as biomarkers, whereas the XWP group was characterized by the genera *Wickerhammomyces* and *Cyberlindnera*. These different microorganisms provide the possibility for unique flavor formation in the different hams (Figures 4C,D).

### 3.5 Network analysis of microbial communities

**Figure 5:**
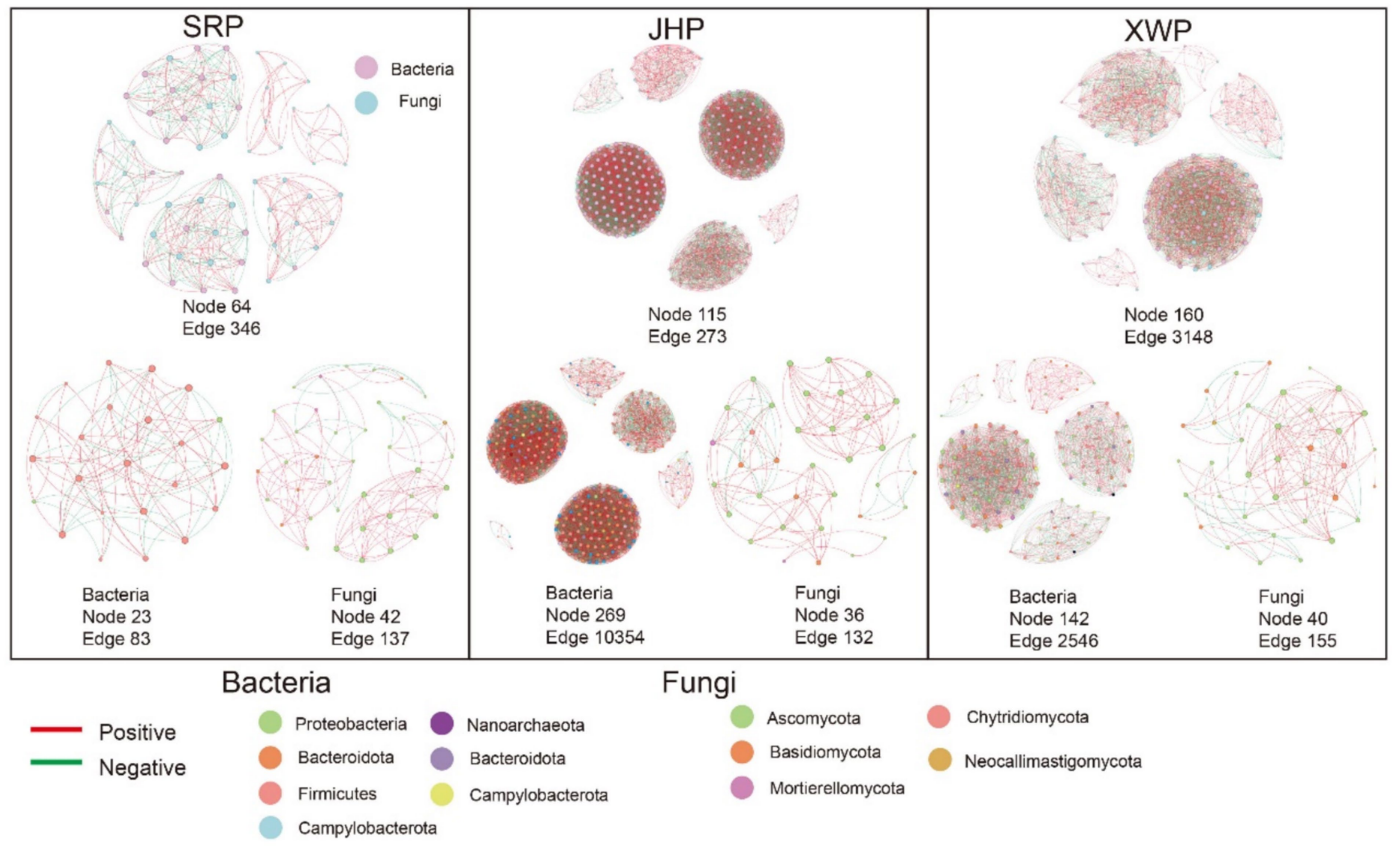
Network analysis based on Spearman correlation. Network interactions between fungi and bacteria were determined between each group and within the fungal and bacterial communities (*r* > 0.6, *p* < 0.05).

The microbial communities were predominantly composed of bacteria and fungi. Investigating the interactions within these communities can offer valuable insight into the microecological dynamics. Our focus extended beyond the interactions between fungal and bacterial communities in each type of ham to include the relationships between individual fungi and bacteria. Overall, the bacteria had a substantial influence on the microbial community, whereas fungi exerted less of an impact on the overall microbial community structure. The SRP microorganisms had the lowest number and complexity of interactions among the three types of ham, whereas the microbial communities in XWP showed the strongest correlations. In addition, the fungi and bacteria in JHP and XWP exhibited close relationships to form a complex network of interactions (Figure 5). In summary, the three types of ham had developed their own distinct microbial networks, with the notable differences between SRP and the other two hams likely attributed to local customs and environmental factors.

## 4 Discussion

Ham, a product reliant on microbial fermentation during its production, owes its flavor profile to the composition and interaction of microorganisms (Chen et al., 2021). In this study, there were relatively few shared bacteria and fungi taxa among the different hams, although JHP and XWP shared more types of bacteria, which may be related to their similar regional characteristics. In this study, it was observed that the diversity and richness of bacteria were significantly higher than that of fungi (Figures 1C,D), consistent with findings from research on Panxian, Xuanen, Sanchuan, and Sabah ham (Mu et al., 2019; Zhang et al., 2020; Deng et al., 2021; Lin et al., 2023). Thus, irrespective of the method of ham production, it was evident that bacteria predominantly governed the fermentation process, with fungi playing a lesser role. SRP, a ham produced in Europe, exhibited the lowest levels of diversity and richness compared with the other groups, yet it had higher levels of fungi, suggesting a close association with the region. Principal coordinates analysis results showed significant differences among the three types of ham, which illustrated the influence of regional characteristics on the fermentation community of ham and the subsequent flavor characteristics.

At the phylum level of bacterial, Firmicutes and Proteobacteria were predominate in all three types of ham, with SRP and JHP particularly dominated by Firmicutes, consistent with the findings of Wang et al. (2021) on the bacterial composition of Norden ham. By contrast, XWP was predominantly inhabited by Proteobacteria, possibly due to the distinct humid and hot environment of that region. During the fermentation process, numerous beneficial and pathogenic bacteria emerged. In SRP, *Lactobacillus* and *Tetragonococcus* dominated the fermentation, whereas *Tetragonococcus* was predominant in JHP and *Pseudomonas* in XWP. The microbial composition strongly correlated with the fermentation region and process (Li et al., 2011). *Pseudomonas* includes many opportunistic pathogenic bacteria, such as *Pseudomonas aeruginosa*, which can cause diseases such as pneumonia and sepsis (Jurado-Martín et al., 2021). Therefore, individuals who are infected or immunocompromised should be cautious about consuming XWP to reduce the risk of infections.

The composition of fungi remained relatively consistent, with notable variations primarily involving *Debaryomyces*, a probiotic fermentation yeast (Breuer and Harms, 2006; Angulo et al., 2020). This genus had a consistent fermentative role across the various types of ham, and its proportions potentially correlated with the stage of fermentation. LEfse analysis is a key method for biomarker screening, and *Lactobacillus*, a dominant microorganism in fermentation, is also an important probiotic (Petrova et al., 2017). *Tetragonococcus* is also a common microorganism involved in fermentation (Wei et al., 2023). The JHP group included a significant number of beneficial gut microbiota, such as *Lachnospiraceae*, *Odoribacter*, and *Alistipes*, which are involved in bile acid metabolism and immune regulation (Parker et al., 2020; Ghosh et al., 2022; Yan et al., 2023). However, it also harbored certain pathogenic bacteria, such as *Staphylococcus*, which was potentially linked to the fermentation process. Therefore, consumption of this type of ham should be approached with caution, especially with consideration of the health status of consumers. *Blautia* was identified as a biomarker in the XWP group and is recognized for its antibacterial and anti-inflammatory properties, making it a potentially beneficial component of the gut microbiota (Liu X. et al., 2021). *Pseudomonas*, however, as a potential pathogen, necessitates thorough disinfection measures during cooking. In the fungal community, fermentation fungi such as *Kurtzmaniella* and *Wickerhamomyces* dominated in SRP, while *Wickerhamiella* and *Cyberlindnera* were dominant in XWP. These distinct fermentation groups have an important role in shaping the unique flavor profiles of each type of ham. Therefore, choosing the appropriate ham for consumption can have beneficial effects on gut microbiota.

Microbial interaction networks are crucial for investigating the interactions among diverse microorganisms within microbial ecosystems (Hassani et al., 2018). The interactions among bacteria shape the overall microbial interaction network and are likely attributed to the diverse functions bacteria perform during the fermentation process (Ma et al., 2022). This phenomenon is closely linked to food spoilage during fermentation. As depicted in Figure 5, notable differences in microbial interactions were present between JHP and XWP hams, two locally produced hams in China, compared with the SRP ham. Specifically, the bacterial relationships in the Chinese-produced hams exhibited closer associations, with tightly interconnected microorganisms that maintain functional stability and resist the proliferation of spoilage bacteria (Chen et al., 2022). This contributes to stabilizing the fermentation process and enhancing the taste of food. Furthermore, varying import and export standards among different countries can also contribute to the occurrence of this phenomenon.

## 5 Conclusion

In conclusion, ham, a widely consumed food globally, was analyzed in this study to compare the microbial communities present in hams from three distinct regions with distinct flavors. The analysis revealed that the bacterial abundance in the microbial community surpassed that of fungi. In addition, ham samples from similar regions shared a higher number of microorganisms. Each of the three types of ham exhibited unique microbial communities with significant differences among them. Notably, *Lactobacillus* and *Tetragonococcus* from the Firmicutes phylum were identified as biomarkers for the SRP group. In the JHP group, biomarkers included *Odorobacter*, *Alistipes*, *Staphylococcus*, *Akkermansia*, and others from the Bacteroidota phylum. The biomarkers of the XWP group were *Pseudomonas*, *Blautia*, and *Bacteroides* from Proteobacteria. Microbial network analysis revealed that bacteria had a more dominant role in the overall microbiota than fungi. The two domestically produced microbial networks in the hams from China showed greater similarity, with tighter associations between microorganisms. These findings may offer insights into the development of rich flavors, the reduction of foreign microbial invasions, and the stability of taste. This study provides a novel microbial perspective on the formation of specific microbial communities in ham, with implications for future quality improvements.

## Data Availability

All of the data supporting this study are available in the NCBl repository, accession number is PRJNA1227238.
